# Deep soft-tissue massage applied to healthy calf muscle has no effect on passive mechanical properties: a randomized, single-blind, cross-over study

**DOI:** 10.1186/s13102-015-0015-8

**Published:** 2015-09-21

**Authors:** Daniel Thomson, Amitabh Gupta, Jesica Arundell, Jack Crosbie

**Affiliations:** 1 School of Science and Health, Western Sydney University, Sydney, Australia; 2 Liverpool Hospital, Liverpool, NSW 2170 Australia

**Keywords:** Tissue mechanics, Massage, Calf muscle, Range of motion, Ankle

## Abstract

**Background:**

Massage is often applied with the intention of improving flexibility or reducing stiffness in musculotendinous tissue. There is, however, a lack of supporting evidence that such mechanical effects occur. The purpose of the study was to investigate the effect of massage on the passive mechanical properties of the calf muscle complex.

**Methods:**

Twenty nine healthy volunteers aged between 18 and 45 years of age had their calf muscle compliance and ankle joint dorsiflexion range of motion (ROM) measured using an instrumented footplate before, immediately and 30 minutes after a ten minute application of deep massage or superficial heating to the calf muscle complex. Repeated measures analysis of variance was used to determine differences between testing sessions and the types of intervention. Reliability testing for the measurement method was conducted using analysis of variance both within and between testing sessions.

**Results:**

There was no significant change in calf muscle stiffness or ankle dorsiflexion range of motion with or without the application of calf massage. Inter- and intra-session reliability were very high, ICC > 0.88 (p < 0.001).

**Conclusions:**

Although individuals’ perception of a change in tissue characteristics following massage has been reported, there was no evidence that soft tissue massage led to a change in the passive mechanical properties of the calf muscle complex. The findings of this study suggest that the use of massage to increase tissue flexibility prior to activity is not justified.

## Background

Massage has been used therapeutically for millennia and involves the manual application of pressure to the soft tissues with the intention of providing beneficial mechanical, physiological, neurological and psychological effects [1]. Evidence supporting these benefits is sparse and there has been little investigation of the mechanical effects of massage, despite its familiar synonym of “soft tissue mobilization”. This is of particular relevance because massage is commonly applied to healthy individuals prior to, or after, undertaking sporting activity, possibly assuming that there are mechanical, rather than therapeutic, effects [2] which decrease injury risk, improve recovery following performance and increase flexibility of the soft tissues [1, 3]. There is, however, little empirical evidence supporting such claims and only small effects have been found with respect to soft tissue flexibility [2, 4] or recovery following exercise [5–7].

Assessment of joint range of motion (ROM) has been used as a proxy for muscle tendon unit (MTU) compliance [2, 8], but does not describe the loading capacity of the soft tissues as they are extended through range. The end of joint ROM may be affected by factors other than the MTU, including ligament tension and intra-capsular resistance to movement. Stiffness, determined as the change in force per unit of change in MTU length [9] and calculated over the total joint ROM, provides a measure that better represents changes in tissue mechanics following massage, as it accounts for changes in the force required to deform the tissue as well as the absolute magnitude of tissue deformation [9, 10].

Continuous measurement of passive stiffness in the calf muscle complex throughout the ankle joint ROM has reliably been obtained using an instrumented footplate which is rhythmically oscillated within the limits of dorsiflexion and plantarflexion. This device records the applied torque and angular displacement of the ankle joint [11, 12]. The use of such an instrumented footplate affords a method to determine whether massage has a passive mechanical effect on the calf muscle complex and thereby enhance our understanding of the clinical utility of massage.

There have been contrasting findings regarding the effect of massage on muscle blood flow and temperature, with only one study finding an increase in local blood flow [13] while other studies have found no change to either blood flow or muscle temperature [6]. Reduction in motor neuron excitability following massage has been observed [14, 15], with a greater reduction in the H-reflex response found with increasing depth of massage [14] possibly inducing muscle relaxation [15]. Whilst there is variable evidence surrounding the potential physiological mechanisms of massage, the purported mechanical mechanisms are unclear.

The purpose of this study was to determine whether massage, applied to healthy individuals, changes the passive mechanical properties of the calf muscle complex, specifically, the stiffness of the MTU through range and the extensibility of the MTU as indicated by the available dorsiflexion range of the ankle.

## Methods

### Design

**Fig. 1:**
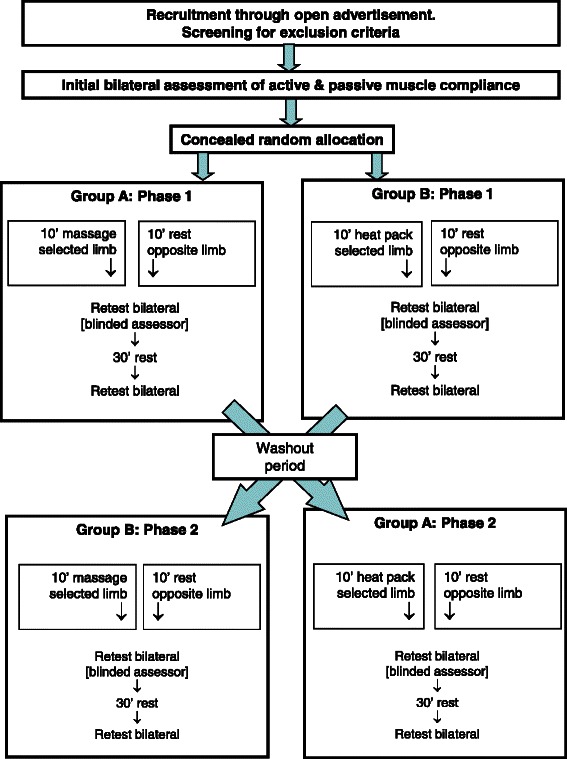
Study design

The biophysical effects of massage may include vascular or sensory changes [13, 15], therefore a comparator in the form of was applied over the calf muscles in an attempt to assess any mechanical effects due to such vascular and sensory changes. A randomised cross-over design was used to investigate the effects of massage and superficial heating on the passive mechanical characteristics of the calf muscle complex [Fig. 1]. A wash-out period of at least ten days was interposed between the two interventions.

### Participants

A total of 29 unimpaired participants (17 male, 12 female) were recruited via open advertisement. Participants were aged between 18 and 45 years of age (mean 22, SD 5) to ensure skeletal maturity and the absence of age-related changes, both of which have been shown to affect MTU stiffness [9, 16]. Exclusion criteria were the presence of any current or recent (<6 months) lower leg injury on either side, a history of significant vascular or neuromuscular illness or impairment affecting the lower limbs (surgical reconstruction, arthritis, stroke, etc.) or skin conditions contraindicating massage. Participants with a body mass index (BMI) >30 kg.m^−2^ were excluded as the leg positioning of obese subjects when measuring stiffness has been reported as problematic [17]. Volunteer participants met all criteria and had a mean BMI of 22.9 (SD 3.3). Written and informed consent was obtained and the rights of the participants were protected at all times. The project received Institutional Ethics Committee approval (University of Western Sydney HREC: #H10544).

### Instrumentation

**Fig. 2:**
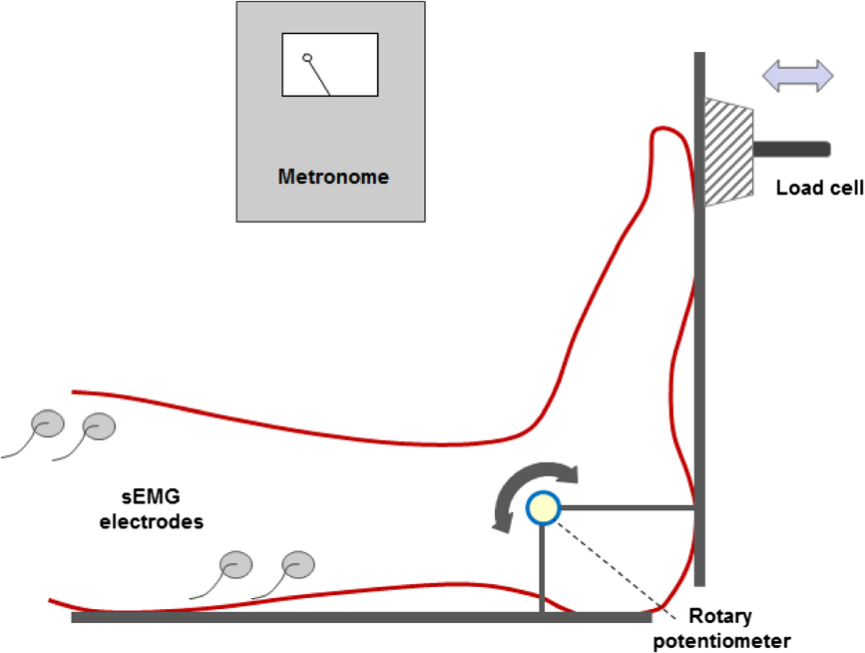
Experimental setup indicating footplate system with the lateral malleolus aligned to the axis of rotation of the rotary potentiometer. Surface electromyographic signals were recorded from the tibialis and soleus muscles. Oscillation of the footplate was synchronized with the audible beat of the metronome and the manually applied force was measured by the load cell

Passive stiffness measures were obtained using a custom-built, hinged, footplate, instrumented with a load cell 1 and potentiometer 2 to record torque and angular displacement and identical to that previously described and validated for measurement of passive ankle stiffness [11, 12] [Fig. 2]. Force and angle data were recorded at a sampling frequency of 200 Hz and stored on a personal computer 3. Surface electromyography (sEMG) signal 4 of the tibialis anterior and soleus muscles were recorded [18] to monitor muscle activity and ensure that processed data were not affected by agonist or antagonist muscle activity during passive ankle motion, which could assist or resist motion of the footplate.

### Testing Procedures

The order of massage or superficial heating of the calf muscle and of the leg treated were randomly selected with the contralateral limb as a resting control. Following the washout period, the alternative intervention was applied to the initial control limb and the contralateral limb acted as the control for the second intervention. To investigate both immediate and persisting effects of the interventions, measures of MTU passive mechanical characteristics were taken prior to, immediately following and 30 minutes after each intervention [Fig. 1]. The order of testing was randomized at each interval. The intervention and the side to which it had been applied was concealed from the assessor performing the measures of by covering both limbs using loose long pants. The fact that the opposite limb was treated in the second intervention was also not disclosed.

Participants remained in a reclined, seated position throughout testing and during the application of each intervention to minimize the effect of confounding variables. Testing was performed by the primary investigator rhythmically oscillating the footplate at a cadence of 0.5 Hz as monitored by an audible metronome. The selected frequency corresponded to ankle dorsiflexion during stance [19]. Fifteen cycles of continuous dorsiflexion-plantarflexion were performed for each test on each side with the knee in both a fully extended position and flexed to 30°. The two positions were used to explore the relative effect of the interventions on the deep and superficial components of the calf complex [20].

### Interventions

Massage of the calf complex was performed for 10 minutes and consisted of petrissage (kneading) strokes, with linking effleurage, applied distal to proximal over the belly of the calf muscle complex. The massage intervention was administered by one of two qualified and experienced physical therapists. To ensure consistency of treatment between therapists, along with participant comfort and safety, participants were asked to report their comfort level as ‘completely tolerable’, ‘strong but tolerable’, ‘uncomfortable’, or ‘intolerable’. The intensity was maintained at a ‘strong but tolerable’ level for consistency and efficacy.

Following screening for thermal sensitivity and being warned of the danger of burns, a superficial heat pack was applied over the belly of the calf muscle complex. The level of heating was adjusted to provide a ‘distinctly warm, but comfortable and even’ heating. Both interventions were provided by an experienced and registered physical therapist.

### Reliability

The reliability of the measurement system was assessed using data from the first 12 subjects. Intra-session reliability was determined by comparing measurements from the control side at the pre-intervention, post-intervention and 30 minute time points. Inter-session reliability was determined by comparing pre-intervention trials on each side at the initial session and that conducted after the wash-out period. Data from both knee positions were collected.

### Data processing and analysis

Of the 15 cycles recorded for each limb in each testing session, the initial two cycles were discarded to exclude possible thixotropic effects [21]. For each remaining cycle, the applied torque values were calculated using the product of force and perpendicular distance between the point of force application and the axis of rotation of the footplate (aligned to the mid-point of the lateral malleolus). These torque values were then scaled by dividing by the subject’s body mass (Nm.kg^−1^); this permits comparison of data across subjects of widely different body size. Ankle dorsiflexion and plantarflexion excursion (degrees) were determined relative to the neutral ankle joint position. If either sEMG signal was above baseline then that trial was discarded ensuring that all measures (torque and angular displacement) were representative of the passive application of force through the ankle by the primary investigator. The remaining cycles were ensemble averaged for further processing.

Intra- and inter-session reliability was determined using intra-class correlation coefficients for ankle dorsiflexion angles resulting from applied torque values of 0.1, 0.15 and 0.2 Nm.kg^−1^ and for the torque values required to move the ankle to 0°, 5° and 10° of dorsiflexion. Visual inspection of measurement consistency utilised Bland-Altman plots [22].

Dependant variables included ankle dorsiflexion position at consistent applied torque values (0.1, 0.15 and 0.2 Nm.kg^−1^) and calf stiffness through ankle dorsiflexion between torque values of 0.1 to 0.2 Nm.kg^−1^computed from the relationship [12]:

$$ \tau = {\mathrm{e}}^{\mathrm{k}.\theta } $$

where *τ* represents applied torque, *k* is the coefficient of stiffness and *θ* is the ankle dorsiflexion angle.

A repeated measures ANOVA was performed to determine significant differences between interventions, with post-hoc analysis using a least significant difference (LSD) test. Statistical significance was accepted at alpha less than 0.05. Independent analyses were conducted for the knee extended and flexed positions for each of the dependant variables.

## Results

There were no statistical or functionally significant differences in ankle dorsiflexion position at the pre-determined torque values between conditions (massage, heat, control) at any time interval or in either the knee extended or knee flexed positions [Table 1]. Similarly, there was no significant difference in dynamic stiffness (*κ*) over the range of applied torque from 0.1 to 0.2 Nm.kg^−1^ across time intervals or between interventions [Table 1].

**Table 1:** Angular displacement (degrees) at each of three applied ankle dorsiflexion torques, and stiffness coefficients between 0.1 and 0.2 Nm · kg^−1^ measured in knee extended and flexed positions (mean (SD))

		Knee extended				Knee flexed			
		0.1 Nm.kg^−1^	0.15 Nm.kg^−1^	0.2 Nm.kg^−1^	Stiffness coefficient (*k*)	0.1 Nm.kg^−1^	0.15 Nm.kg^−1^	0.2 Nm.kg^−1^	Stiffness coefficient (*k*)
Pre-treatment	Heat pack	4.4 [5.7]	10.6 [5.9]	19.0 [6.3]	21.0 [3.7]	14.3 [5.7]	22.0 [6.3]	31.4 [6.9]	23.1 [4.4]
	Heat control	3.8 [5.8]	10.2 [6.1]	19.3 [6.6]	21.6 [6.4]	13.6 [5.3]	21.7 [5.5]	31.8 [6.8]	23.5 [6.4]
	Massage	3.6 [5.7]	10.1 [5.9]	19.1 [7.1]	21.9 [5.5]	13.4 [6.6]	21.2 [6.0]	30.9 [5.9]	24.3 [6.5]
	Massage control	3.6 [6.2]	9.7 [6.5]	18.3 [6.9]	21.4 [2.9]	14.4 [7.3]	21.6 [7.5]	30.6 [7.2]	23.4 [3.3]
Post-treatment	Heat pack	5.4 [5.5]	11.5 [5.4]	19.7 [5.9]	21.6 [3.7]	13.7 [6.9]	21.4 [7.2]	30.7 [7.6]	23.7 [4.2]
	Heat control	4.1 [5.2]	10.6 [5.5]	19.3 [6.5]	21.7 [6.0]	13.6 [5.9]	21.5 [6.0]	31.3 [7.0]	24.1 [6.8]
	Massage	4.4 [5.9]	11.0 [6.1]	19.7 [7.7]	22.6 [5.9]	14.0 [7.0]	22.2 [7.0]	32.2 [7.5]	24.2 [6.8]
	Massage control	4.5 [5.8]	10.6 [6.0]	19.1 [6.6]	21.7 [3.5]	14.3 [7.3]	21.8 [7.3]	30.8 [6.8]	23.6 [3.6]
30’ post treatment	Heat pack	4.4 [5.3]	10.5 [5.4]	18.8 [5.9]	21.2 [3.7]	14.5 [6.6]	22.0 [7.1]	31.1 [7..6]	23.5 [4.6]
	Heat control	4.3 [5.3]	10.6 [5.9]	19.4 [6.7]	21.8 [5.9]	14.2 [5.9]	21.9 [6.1]	31.5 [7.0]	23.9 [6.7]
	Massage	3.8 [6.6]	10.4 [7.1]	19.1 [8.3]	22.3 [6.8]	13.3 [6.6]	21.7 [6.7]	31.6 [7.5]	24.0 [6.7]
	Massage control	4.4 [5.7]	10.5 [6.0]	18.8 [6.7]	21.5 [3.7]	14.6 [6.9]	22.1 [7.2]	30.9 [7.4]	23.7 [4.4]

It was incidentally noted that the difference in angular displacement brought about by an applied torque of 0.2 Nm.kg^−1^ with the knee extended and flexed was relatively uniform at each time interval and equated to a mean of 12° (SD 4), indicative of the difference between tension applied to the soleus muscle alone compared to the calf complex as a whole [Table 1].

Reliability of the measurement protocol was shown to be high for both intra-session and inter-session tests for both sides and in both conditions of knee flexion-extension [Table 2]. A median of 11 trials per participant per test were available for derivation of outcome variables. The frequency of the footplate oscillation was 0.501Hz (SD 0.005), matching the target frequency.

**Table 2:** Intraclass correlation (ICC) values for tests of reliability

Variable	Side	Knee extended		Knee flexed	
		Inter-session ICC	Intra-session ICC	Inter-session ICC	Intra-session ICC
Angle @ 0.1 Nm.kg^−1^	Right	0.990	0.959	0.992	0.980
	Left	0.995	0.982	0.989	0.935
Angle @ 0.15 Nm.kg^−1^	Right	0.994	0.973	0.986	0.993
	Left	0.997	0.990	0.995	0.982
Angle @ 0.2 Nm.kg^−1^	Right	0.990	0.945	0.989	0.987
	Left	0.998	0.983	0.997	0.979
Torque @ 0°	Right	0.984	0.905	0.965	0.894
	Left	0.979	0.931	0.944	0.897
Torque @ 5°	Right	0.994	0.959	0.967	0.889
	Left	0.975	0.928	0.971	0.913
Torque @ 10°	Right	0.994	0.981	0.971	0.918
	Left	0.974	0.916	0.986	0.925

**Fig. 3:**
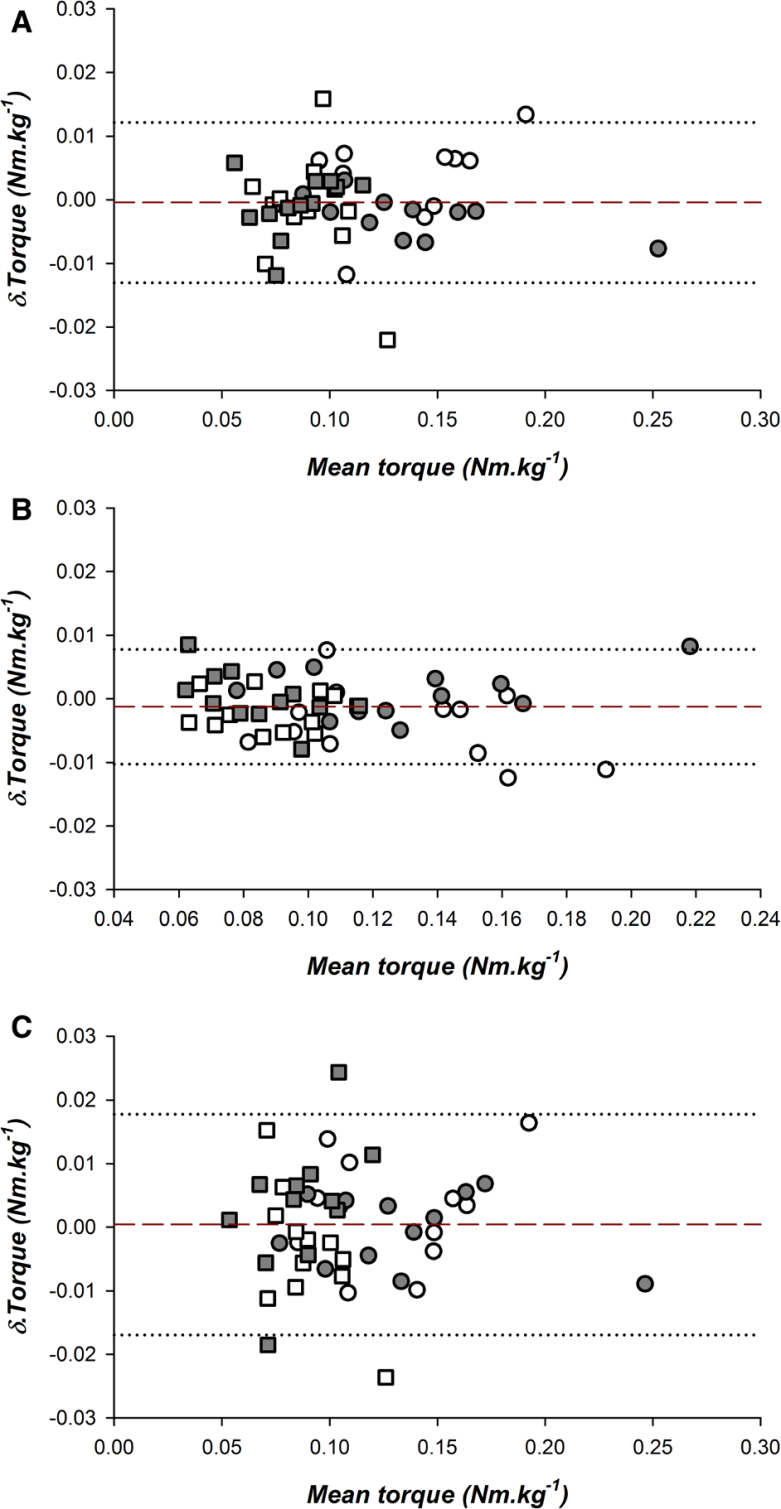
Reliability of instrumentation. Bland-Altman plots (mean and 95 % limits of agreement) for torque values required to dorsiflex the ankle to 10 degrees. Comparisons are made for inter-session trials (A = initial trial compared to trial at 10 minutes; B = trial at 10 minutes compared to trial at 30 minutes; C = initial trial compared to trial two weeks later). Open circles represent right side with knee extended; filled circles represent left side with knee extended; open squares represent right side with knee flexed; filled squares represent left side with knee flexed

Bland Altman plots [Fig. 3] demonstrated for intra-session measures that the mean difference between pretest and immediate post-test was −0.43 · 10^−3^ Nm.kg^−1^ with limits of agreement ± 0.0126 and the mean difference between the immediate post-test and 30 minutes was −1.23 · 10^−3^ Nm.kg^−1^ (limits of agreement ± 0.009). Inter-session mean difference was 0.42 · 10^−3^ Nm.kg^−1^ (limits of agreement ± 0.017).

## Discussion

This study clearly demonstrated that massage did not affect the passive mechanical characteristics of the calf muscle. Neither calf stiffness calculated across a range of applied torques, nor ankle angular excursion at specific torque values changed after massage. Although it has been hypothesized that massage will decrease passive stiffness of the MTU and thereby increase the available range of motion of the joint it crosses [1, 2] the current study provides empirical evidence that massage has no effect on the resting calf muscle. It is plausible that there are other physiological or psychological effects due to massage, however these do not impact on the passive mechanical characteristics of the MTU.

The current study sampled healthy participants and it is possible that massage may be more effective when there are transient or longer-term changes in the compliance of the MTU. Or it may be that massage is more effective following vigorous physical activity, including muscle soreness due to eccentric activity, where possible changes may be found in muscle stiffness. These options remain to be explored. Some studies have indicated positive benefits of massage on delayed onset muscle soreness [5–7], although the possibility of purely psychological benefits cannot be excluded. Nevertheless, it would be logical to apply the current protocol to participants where there are demonstrable alterations to calf muscle stiffness.

This study highlights the need to be able to accurately evaluate the possible effects on mechanical characteristics of the MTU. The current study used an instrument which has previously been validated for this type of investigation [10, 12] and demonstrated very high test-re-test reliability with only small variance in recorded values of calf stiffness and ankle joint ROM within and between sessions. The current results for torque/angle relationships were consistent with those previously reported using similar instrumentation in a comparable population [12]. There is, therefore, reason to believe that these results are representative of the passive mechanical characteristics of the calf muscles and sensitive enough to detect any clinically relevant changes.

The duration of massage applied in this study was substantially in excess of previous investigations of the effects of massage on ankle ROM [2, 4] and more closely matched clinical practice. The current study involved blinding of the assessor to intervention and the insurance that ankle joint displacement was exclusively a function of the applied torque. This method contrast to some earlier studies [4, 8] in which the change in ROM may have been attributed to other factors such as an inconsistently applied torque or increased tolerance to stretch. Further, participants were not exposed to any movement or weight-bearing activity over the course of testing, which might affect their tissue compliance.

The inclusion of superficial heating as a comparator intervention was intended to assist in differentiating changes notionally due to increased temperature and blood flow to the underlying tissue, possibly affecting the mechanical characteristics. However, the absence of any change associated with either intervention indicated that neither of these interventions had a clinically significant effect on the passive mechanical characteristics of the calf muscle complex.

The dynamic stretching at 0.5 Hz and the range of torque values applied to the ankle compare with the cyclical cadence and kinetics of ankle motion during walking [19]. Therefore, the lack of statistically significant differences represents a lack of functionally relevant effects on calf stiffness and ankle joint ROM from either massage or heat. Neither massage or superficial heating demonstrate any clinically meaningful effects on MTU stiffness in healthy, unimpaired subjects. This leads us to believe that the use of massage prior to activity, as a means of increasing tissue flexibility, is unjustified.

## Conclusions

The null hypothesis, that neither massage nor superficial heating would alter the passive mechanical properties of the calf muscle complex was supported. No significant effects were observed for dynamic stiffness or extensibility of the calf muscle complex in either position of testing. Our data support the assertion that, in a healthy population, deep, soft-tissue massage does not alter passive mechanical characteristics of the calf muscle complex.

## Abbreviations

ANOVA: Analysis of variance
BMI: Body mass index
MTU: Muscle tendon unit
ROM: Range of motion
sEMG: Surface electromyography

## Footnotes

1 Xtran 450 N load cell, Applied Measurement, Bankstown, NSW;

2 Rotary potentiometer, Jaycar, Rydalmere, Sydney, NSW;

3 National Instruments, North Ryde, Sydney, NSW;

4 Zerowire EMG system, Cometa Inc., Milan, Italy.
